# Early Joint Affections in Syphilis

**Published:** 1882-09

**Authors:** 


### ORIGINAL TRANSLATIONS AND ABSTRACTS.

### EARLY JOINT AFFECTIONS IN SYPHILIS.

Systematic works on syphilis make generally but a passing mention of the joint affections, occurring contemporaneously with the early eruptive manifestations of the disease. In a recent paper (*New Orleans Medical and Surg. Journal*, August, 1882,) Dr. George H. Rohé reports a case, and adds a bibliographical summary of the literature of the subject. According to Dr. Rohé, Lancereaux credits Babington with having first clearly recognized syphilitic joint affections in the so-called secondary period of the disease. Lancereaux adds two cases in full, and refers to several others observed by him. The symptoms have a great resemblance to rheumatism. In both diseases the joints are swollen, red and painful. In syphilis, however, the swelling and redness are less in degree than in rheumatism. The patients describe the pains as breaking or tearing, subject to nocturnal exacerbations, and slightly increased on movement. The knees, wrists and elbows are said, by Lancereaux, to be most frequently affected. Rarely is a single joint attacked, but, as in rheumatism, the arthropathies are multiple. Fever, if present, is generally much less in the syphilitic arthropathy; in fact, the inflammation, if any be present, is decidedly subacute. Keyes, Bumstead and Taylor refer briefly to the affection as if it were well known, but, on the other hand, Zeisel states, that aside from the rheumatoid pains, which are very frequent in the early period of the disease, he has seen very few cases of joint affection in syphilitic patients, although his opportunities for observation were exceptional. He seems strongly inclined to doubt the ethiological relation of the disease to the local morbid process. Vaffier states, that according to his observation, early joint affections are much more frequent than is generally believed. He asserts that in China and Japan, in which this writer served as an officer in the French navy, a rheumatic joint affection occurs in nearly every case in the secondary stage of syphilis. Voisin testifies to the prevalence of syphilitic arthrophatics in the secondary period. They may occur as simple painful joints, or as acute and subacute inflammations. More recently Marcek has contributed an excellent bibliographical and clinical paper on the subject. In other recent works, the subject is either not mentioned or is passed over in a very perfunctory manner. The treatment, in addition to the general anti-syphilitic measures—mercury in small doses, 1-40–1-25 grain of bichloride and iodide of potassium, should comprise applications. Among these the best are probably mercurials. Mercurial plaster or a five *per cent.* ointment of the oleate giving the best results. Where the pain is severe, the oleate of mercury and morphia will give most satisfaction.

